# Preparation of a new composite combining strengthened β-tricalcium phosphate with platelet-rich plasma as a potential scaffold for the repair of bone defects

**DOI:** 10.3892/etm.2014.1912

**Published:** 2014-08-18

**Authors:** CHENGGONG WANG, DA ZHONG, XING ZHOU, KE YIN, QIANDE LIAO, LINGYU KONG, ANSONG LIU

**Affiliations:** Department of Orthopedics, Xiangya Hospital, Central South University, Changsha, Hunan 410008, P.R. China

**Keywords:** tricalcium phosphate, platelet-rich plasma, tissue engineering, bone defect, bone marrow stromal cells

## Abstract

β-tricalcium phosphate (β-TCP) and platelet-rich plasma (PRP) are commonly used in bone tissue engineering. In the present study, a new composite combining strengthened β-TCP and PRP was prepared and its morphological and mechanical properties were investigated by scanning electron microscopy (SEM) and material testing. The biocompatibility was evaluated by measuring the adhesion rate and cytotoxicity of bone marrow stromal cells (BMSCs). The strengthened β-TCP/PRP composite had an appearance like the fungus *Boletus kermesinus* with the PRP gel distributed on the surface of the micropores. The maximum load and load intensity were 945.6±86.4 N and 13.1±0.5 MPa, which were significantly higher than those of β-TCP (110.1±14.3 N and 1.6±0.2 MPa; P<0.05). The BMSC adhesion rate on the strengthened β-TCP/PRP composite was >96% after 24 h, with a cell cytotoxicity value of zero. SEM micrographs revealed that following seeding of BMSCs onto the composite in high-glucose Dulbecco’s modified Eagle’s medium culture for two weeks, the cells grew well and exhibited fusiform, spherical and polygonal morphologies, as well as pseudopodial connections. The strengthened β-TCP/PRP composite has the potential to be used as a scaffold in bone tissue engineering due to its effective biocompatibility and mechanical properties.

## Introduction

Bone defects caused by infection, trauma or tumors remain intractable clinical issues. Study of implantable biomedical materials is particularly important in the treatment of large bone defects where self-repair of the human body is extremely difficult. Currently, there are three main treatment methods for repairing bone defects: autogenous bone grafts, allogeneic bone grafts and implantation of biomedical materials (1). However, none of these methods are perfect. For example, sources of autogenous bone grafts are limited and additional trauma to the patient cannot be avoided during the procedure. Although allogeneic bone grafts and biomedical materials are easier to obtain, other problems exist, including the risk of spreading disease, transplantation-associated immune reactions and generally poor biodegradability and long-term outcomes.

The rapid development of bone tissue engineering has generated new theories and technologies for repairing bone defects in which seed cells, scaffolds and osteogenic factors are considered to be the basic components for constructing tissue-engineered bone (2,3). The scaffold material acts as an artificial extracellular matrix (ECM) and provides support for cell adhesion, growth, proliferation, metabolism and the formation of new bone tissue. Currently, artificially synthesized inorganic materials (including hydroxyapatite and calcium phosphate) and polymer materials [including polylactic acid, polyglycolic acid (PGA), polyphophazenes and chitosan] may be used as scaffolds for bone tissue engineering (4–6). However, all of these materials have certain limitations in biocompatibility, biodegradability or mechanical matching (7–10). Therefore, additional research is required to investigate other types of composite materials.

In the present study, a new gel composite combining strengthened β-tricalcium phosphate (β-TCP) with platelet-rich plasma (PRP) was prepared. Its potential application as a scaffold for repairing bone defects was evaluated by analyzing the compressive strength, cell adhesion and cell cytotoxicity of the material.

## Materials and methods

### Preparation of strengthened β-TCP

The β-TCP was prepared through chemical precipitation as follows: i) 58.82 g (NH_4_)_2_HPO_4_ and 141.69 g Ca(NO_3_)_2_·4H_2_O were dissolved in distilled water, respectively. (NH_4_)_2_HPO_4_ solution was added to the Ca(NO_3_)_2_ solution at a sustained rate of 1.5 ml/min, with the pH value adjusted to 7.0–7.5 by the addition of NH_4_OH solution. The supernatant was removed 24 h following the formation of a white precipitate, which was washed with distilled water until the conductivity of the liquid was <200 μS/cm; ii) The precipitate was transferred to a metal container, compressively molded and calcined at 850°C for 10 h to obtain the β-TCP sample; iii) the precipitate was dried at 120°C and calcined at 850°C for 0.5 h to obtain the β-TCP powder. The β-TCP powder was mixed with 4% hyaluronic acid (HA) nanoparticles and added to distilled water together with 0.5% dispersant polyethylene glycol to obtain a slurry (solid content, 60%). The slurry was milled for 24 h and added to bioglass powder (total solid content, 20%) for additional milling for 2 h. Surfactant Tween 80 (Lantian, Xingtai, China) was added to the slurry, which was subsequently stirred (rotation speed, 500 rpm) to generate foam with a double-leaf stirring blade in a controlled N_2_ atmosphere. The foamed slurry was poured into a plaster cast for molding. Once dried, the molding was sintered by first heating to 570°C at a rate of 2°C/min, maintained at this temperature for 1 h, and subsequently heated to 1,000°C at a rate of 5°C/min, where it was maintained for an additional 1 h. The strengthened β-TCP sample was prepared using this method.

### Preparation of PRP

To prepare the PRP, 10 ml venous blood was collected from the hind limb of a beagle dog and placed in a 15-ml centrifuge tube containing 1 mg sodium citrate. PRP was extracted using a two-step centrifugation method. First, the blood sample was centrifuged at 1,500 rpm for 10 min. The upper layer of plasma and the erythrocytes located within 2 mm of the interface were transferred to another centrifuge tube. Secondly, the sample was centrifuged at 3,600 rpm for 10 min and the upper plasma layer, containing a small number of suspended platelets, was removed. The remaining plasma (~1 ml) was PRP. A total of 10 μl whole blood and 10 μl PRP were drawn from the centrifuge tube with a pipette and diluted to 2 ml by platelet dilution. One drop of the mixture was transferred to a clean counting chamber, incubated at room temperature for 10 min and the cells were counted under a CX22 light microscope (Olympus, Tokyo, Japan) at high magnification (x400). Additional PRP was prepared using this method and stored in a refrigerator at −70°C. The current study, including the animal ethics, was approved by the Ethics Committee of Xiangya Hospital (Changsha, China).

### Preparation of the composite combining strengthened β-TCP and PRP

The strengthened β-TCP samples (cylindrical, diameter, 1.0 cm; height, 4.0 cm) were cut into 0.5-cm slices using a razor blade in an ultra-clean cabinet. The slices were repeatedly washed with saline and sterilized at 180°C for 30 min after drying. The slices were subsequently immersed in PRP until the tricalcium phosphate material was completely enveloped by the plasma. The activating agent (10% calcium chloride solution containing 100 μg/ml bovine thrombin) was added at a ratio of 1:1. The mixture was incubated in a 37°C water bath to form the strengthened β-TCP/PRP gel composite.

### Isolation, cultivation, purification and proliferation of beagle dog bone marrow stromal cells (BMSCs)

A 12-month-old adult beagle dog weighing 10 kg was administered intravenous anesthesia with 3% pentobarbital (1.0 ml/kg) and 3 ml bone marrow was extracted using a sterile technique. The marrow was washed and diluted with phosphate-buffered saline (PBS) and slowly poured over Percoll (1.079 g/ml; Pharmacia, Beijin, China) at a volume ratio of 1:1. The mixture was centrifuged at 2,000 rpm for 15 min, during which time the centrifuged mixture formed four layers in the tube. The second white membrane-like layer was drawn with a syringe, washed with PBS and centrifuged at 1,000 rpm for 5 min. Following removal of the supernatant, Dulbecco’s modified Eagle’s medium-low glucose (DMEM-LG) culture (containing 100 μg/ml penicillin and 100 μg/ml streptomycin) supplemented with 10% fetal calf serum (FCS) was added to resuspend the cells. The cells were seeded in a 25-cm^2^ culture flask at a density of 2×10^5^ cells/cm^2^ together with 4 ml of the culture medium and placed in a CO_2_ cell culture incubator. The medium was changed following the first 48 h of incubation and every three days thereafter. Finally, 0.25% trypsin digestion was performed when the adherent cell confluence reached 80–90%. Serial subculture was performed at a 1:3 dilution. A control was prepared for the adhesion rate assay by adding the BSMC suspension to the composite.

### BMSC adhesion rate and cell cytotoxicity

Based on the method proposed by Kasten *et al* (11), the strengthened β-TCP/PRP gel composite was placed in 24-well culture plates. To the composite was added dropwise 0.2 ml BMSC suspension (concentration, 1×10^5^ cells/ml) and cultured in a humidified cell culture incubator (37°C, 5% CO_2_, saturated humidity). The samples were collected at 4, 8, 12 or 24 h following incubation (n=5 for each time point). The adherence rate (%) = (inoculation cell number - number of cells lost - non-adherent cell number)/(inoculation cell number - number of cells lost) ×100.

BMSCs were collected following the third passage and the density of the cell suspension was adjusted to 1.0×10^5^ cells/l. The cell suspension was seeded onto the strengthened β-TCP/PRP composite through a micropipettor with 50 μl in each bracket to produce a cell-composite complex and incubated in the cell culture incubator. BMSCs with the same density as the cell-composite complex above were plated in wells as the control group and five parallel samples were used for each group. The proliferation rate was measured at 2, 4 and 8 days following seeding using a 3-(4,5-dimethylthiazol-2-yl)-2,5-diphenyltetrazolium bromide (MTT) assay. Briefly, the samples in each well were mixed with 100 μl 5% MTT and cultured for an additional 4–6 h. The liquid in each well was removed and 500 μl dimethyl sulfoxide (DMSO) was added. The cells were incubated for 10 min and 200 μl supernatant from each well was removed and plated in individual wells of a 96-well culture plate. The absorbance was measured at a wavelength of 490 nm using a microplate reader (SpectraMax M5; Molecular Devices, Sunnyvale, CA, USA). The mean absorbance was taken from six wells. The relative growth rate (RGR) of each group was calculated using the following formula: RGR (%) = [mean optical density (OD) value of the experimental group/mean OD value of control group] × 100. The RGR values for each group were converted into six-degree cytotoxicities to assess the toxicity of the materials. These cytotoxicities were on a scale of 0–5 as follows: 0, RGR >1; 1, RGR 0.75–0.99; 2, RGR 0.5–0.74; 3, RGR 0.25–0.49; 4, RGR 0.01–0.24; 5, RGR 0.

### Scanning electron microscopy (SEM) examination of the seeded BMSCs on different scaffolds

The BMSCs were collected following the third passage and the density of the cell suspension was adjusted to 4.0×10^10^ cells/l. The cell suspension was seeded onto strengthened β-TCP and the β-TCP/PRP composite using a micropipettor with 100 μl in each bracket and the cells were incubated in the cell culture incubator for 4 h to allow for cell adherence. Three experimental groups were assessed: i) BMSCs seeded on strengthened β-TCP with low-glucose DMEM culture medium containing 10% FCS; ii) BMSCs seeded on strengthened β-TCP/PRP with low-glucose DMEM culture medium containing 10% FCS and; iii) BMSCs seeded on strengthened β-TCP/PRP with an inducer (high-glucose DMEM culture medium containing 10% FCS, 10^−7^ mol/l dexamethasone, 10 mmol/l β-glycerophosphate and 500 mg/l ascorbic acid).

Following culture of the cells for two weeks, the composite was examined with a SEM (Jeol JSM-6360LV; Jeol, Inc., Tokyo, Japan) to observe cell growth and proliferation on the materials as well as matrix secretion. The steps to prepare the samples prior to SEM observation were as follows: i) cleaning, the samples were immersed in saline for ultrasonic cleaning for 5–10 min; ii) fixing, the samples were fixed with 4% glutaraldehyde for 2 h and subsequently with 1% osmic acid for 2 h; iii) dehydration, gradient dehydration was performed by incubating each of the samples in 50, 70, 90 and 100% acetone for 10 min; iv) substitution, performed by incubating the samples with 50, 70, 90 and 100% isoamyl acetate for 10 min each; v) drying, an HCP2 critical point dryer (Hitachi Ltd., Tokyo, Japan) was used for drying; and vi) coating, Emitech K500X/K550X Sputter targets (Quorum Technologies, Ltd., Laughton, UK) were used to plate gold film onto the samples in a vacuum. Furthermore, the morphologies of strengthened β-TCP and strengthened β-TCP/PRP composite were also examined using SEM.

### Biomechanical properties of β-TCP and strengthened β-TCP/PRP composite

The β-TCP (five samples) and strengthened β-TCP/PRP composite (five samples) were placed vertically in an Instron 8032 universal material tester (Instron, Norwood, MA, USA) for compression analysis with a loading speed of 5 mm/min. The maximum load (maximum external force that the sample was able to bear) and the utmost intensity (calculated as maximum load/contact area) were recorded during the experiment.

### Statistical analysis

Results are presented as mean ± standard deviation and statistical analyses were performed using SPSS software, version 13.0 (SPSS, Inc., Chicago, IL, USA). A double sample Student’s t-test was used to evaluate the differences between groups and a difference was considered to be statistically significant if two-tailed P<0.05.

## Results

### Morphological properties of the strengthened β-TCP and strengthened β-TCP/PRP composite

**Figure 1:**
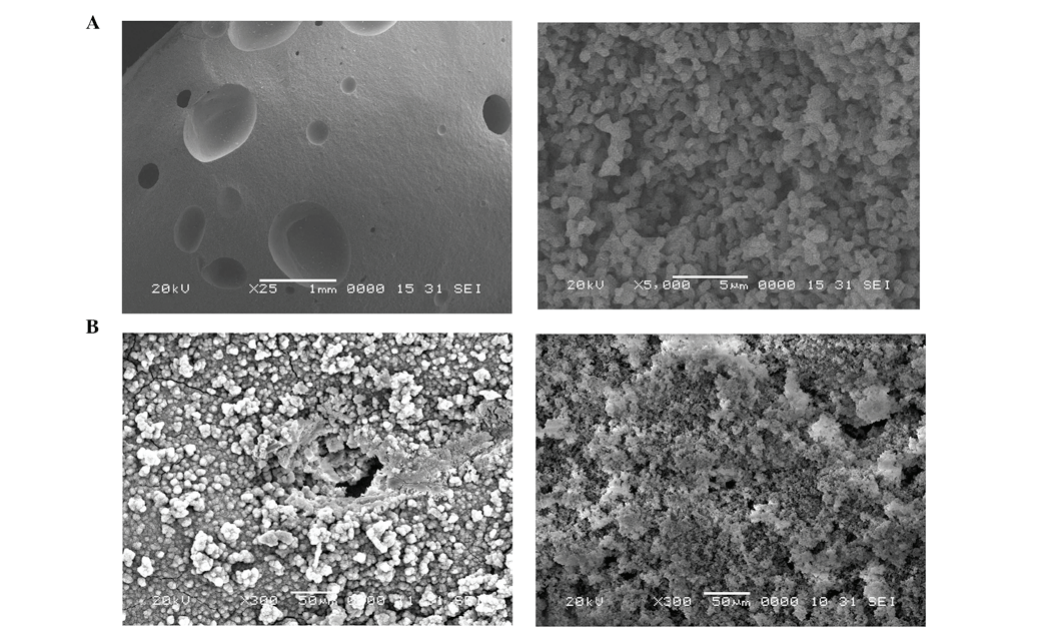
Scanning electron microscopy (SEM) micrographs. (A) Strengthened β-tricalcium phosphate (β-TCP); a white porous material with different pore sizes (100–400 μm) and the presence of micropores distributed with diameters of 1–10 μm, magnification, ×25 (left) and ×5,000 (right). (B) Strengthened β-TCP/platelet-rich plasma (PRP) composite; a kermesinus-like material with PRP gel distributed on the surface and the micropores. The micropores appear smaller as a result of being filled by the PRP particles. Magnification, ×300 for both images.

The concentration of platelets present in the PRP prepared using a two-step centrifugation procedure was calculated to be 1,239.3±124.0×10^9^ cells/l, which was ~8.6-fold higher compared with that of venous blood (143.3±31.9×10^9^ cells/l). Strengthened β-TCP, a white porous material with a variety of pore sizes (range, 100–400 μm), was prepared in the form of a cylinder (diameter, 1 cm; height, 3–4 cm). SEM analysis revealed a granular appearance at high magnification (x5,000; Fig. 1A). The pores on the surface connected with each other and small micropores were distributed with diameters of 1–10 μm. The PRP gel appeared as particulate crystals and was well distributed on the surface as well as the pores of the strengthened β-TCP/PRP composite, giving the material an appearance like the fungus *Boletus kermesinus* (referred to as kermesinus). The micropores on the surface of the composite were observed to be smaller due to the filling of the PRP particles when viewed by SEM at high magnification (x300; Fig. 1B).

### Adhesion rate and cell cytotoxicity of BMSCs grown on strengthened β-TCP/PRP composite

The BMSC adhesion rate on the strengthened β-TCP/PRP composite was 50±2, 80±1, 92±2 and 96±1% after 4, 8, 12 and 24 h, respectively. There were no significant differences in the adhesion rate at any of these time points compared with those in the control (P>0.05). The OD and RGR values of the control and experimental groups are shown in Table I. According to the criteria used in the current study, the cytotoxicity of the strengthened β-TCP/PRP composite was zero.

### SEM examination of seeded BMSCs on different scaffolds

**Figure 2:**
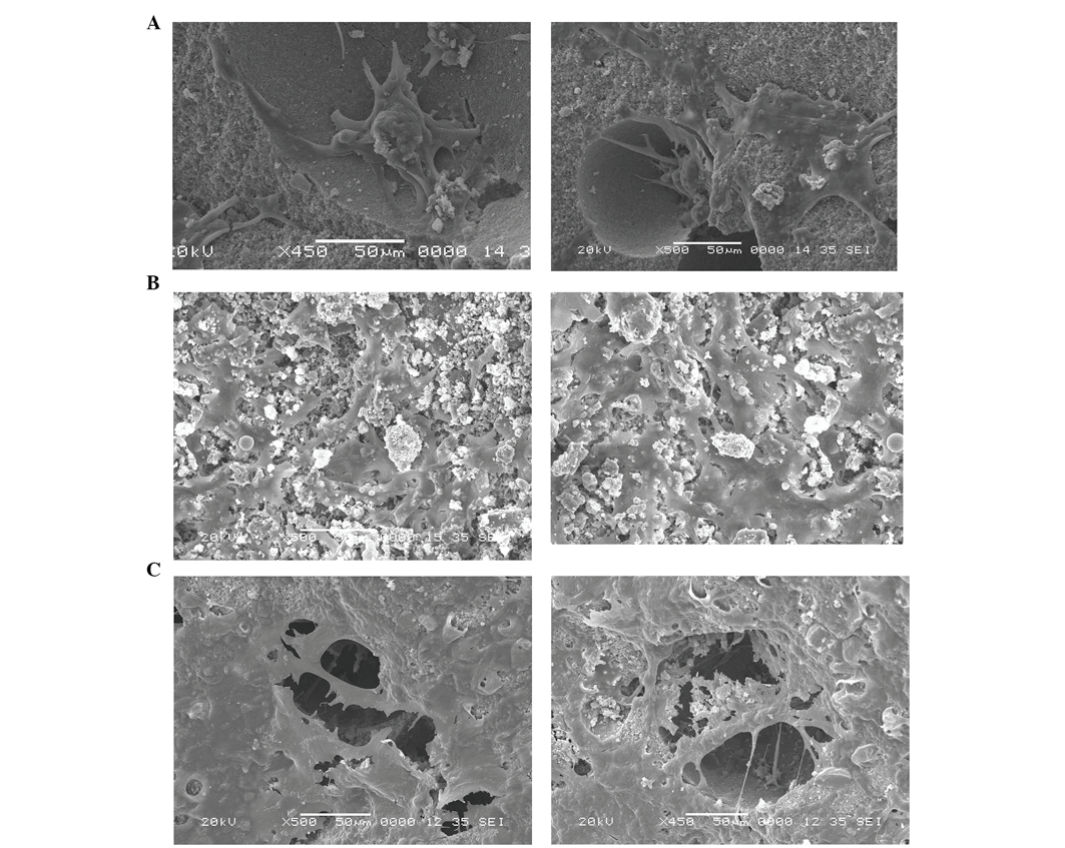
Scanning electron microscopy (SEM) micrographs. (A) Bone marrow stromal cells (BMSCs) seeded on strengthened β-tricalcium phosphate (β-TCP) grown in low-glucose Dulbecco’s modified Eagle’s medium (DMEM) culture. The cells grew slowly and had a fusiform morphology. Magnification, ×450 (left) and ×500 (right). (B) BMSCs seeded on strengthened β-TCP/platelet-rich plasma (PRP) grown in low-glucose DMEM culture. The cells grew faster and exhibited fusiform, spherical or polygonal morphologies. Magnification, ×500 (left) and ×500 (right). (C) BMSCs seeded on strengthened β-TCP/PRP grown in high-glucose DMEM culture. There were a large number of cells on the surface and around the pores, which exhibited fusiform, spherical or polygonal morphologies as well as pseudopodia connections. Magnification, ×500 (left) and ×450 (right).

There was a low number of BMSCs seeded on the strengthened β-TCP grown in low-glucose DMEM culture medium and the cells grew slowly with a fusiform morphology (Fig. 2A). The BMSCs seeded on strengthened β-TCP/PRP in low-glucose DMEM culture medium grew faster and exhibited fusiform, spherical and polygonal morphologies (Fig. 2B). For the strengthened β-TCP/PRP in high-glucose DMEM culture medium, a large number of BMSCs were attached to the surface and around the pores, and exhibited fusiform, spherical and polygonal morphologies. Pseudopodia extending from the cells were observed that connected with each other and covered the pores; the secretion of ECM was also observed (Fig. 2C).

### Biomechanical properties of β-TCP and the strengthened β-TCP/PRP composite

The maximum load and utmost intensity of the strengthened β-TCP/PRP gel composite were 945.6±86.4 N and 13.1±0.5 MPa, respectively, which were significantly higher compared with those of β-TCP (110.1±14.3 N and 1.6±0.2 MPa, respectively; P<0.05). The utmost intensity of the strengthened β-TCP/PRP gel composite was also slightly higher than that of human cancellous bone (4–12 MPa).

## Discussion

The ideal materials for bone tissue engineering scaffolds should have the following characteristics: i) Effective biocompatibility, non-toxic degradation products and no inflammation; ii) suitable pore size for the in-growth of new bone tissue, with an average diameter of 200–400 μm; iii) enough mechanical strength to support new bone tissue with suitable mechanical properties; iv) osteoconductive or osteoinductive effects to promote bone deposition and growth and; v) adjustable biodegradability. In the present study, β-tricalcium phosphate with good physicochemical properties was used as the matrix, bioglass as the binder and nano-HA was used for the dispersal phase to prepare strengthened β-TCP using the physical foaming method. Strengthened β-TCP had mutually-connected large pores and micropores, with a relatively small pore size distribution. The diameters of the large pores ranged between 750 and 850 μm, and a number of micropores with a diameter of ~21 μm were also present on the walls of the large pores. Furthermore, the composite material had a good biocompatibility and mechanical properties; therefore, it has the potential to be used as a scaffold in bone tissue engineering to repair bone defects.

Artificial polymers and bioactive ceramics are commonly used scaffold materials for bone tissue engineering. In a study by Breitbart *et al*, PGA fiber scaffolds containing rabbit periosteal osteoblasts completely repaired bone defects (diameter, 1–5 mm) in a rabbit skull after 12 weeks (12). Sachlos *et al* increased the hydrophilicity of scaffolds by introducing chemical functional groups to their surface; local aseptic inflammation was observed in certain patients following implantation of these scaffolds (13). HA and tricalcium phosphate (TCP) are common bioactive ceramics used in tissue engineering. In a study by Morishita *et al*, BMSCs forced to differentiate into osteoblasts on HA ceramics revealed a strong osteogenic ability and repaired bone defects within six months (14). However, the brittleness and low degradation rate of HA ceramics limits their application in bone tissue engineering to a certain extent. TCP has effective physicochemical properties, biocompatibility and biodegradability, which makes it an efficient bone graft material that is able to promote new bone formation and conduction, and repair bone defects. Harris and Cooper loaded human mesenchymal stem cells onto cubes of coral calcium carbonate-derived apatite, bovine bone-derived apatite, synthetic HA/TCP (60/40%) or synthetic HA/TCP (20/80%) and placed them into the dorsal regions of mice with severe combined immunodeficiency (SCID) for five weeks (15). Histomorphometric analysis of bone development within the cubes revealed an absence of bone formation within the coral-derived and bovine bone-derived apatites. However, bone formation within the synthetic HA/TCP scaffolds was revealed to comprise 8.8 and 13.8% of the total tissue present for the 60:40 and 20:80% materials, respectively (15). TCP has certain disadvantages, including an excessive degradation rate *in vivo* as well as high rigidity and brittleness. For example, in a study by Wiltfang *et al*, 3.5–4.7 ml cancellous bone defects were established on the two sides of the tibial head in seven Goettingen minipigs (16). After 16 and 28 weeks post-filling of the defects with β-TCP, the degradation rate was 60 and 80%, respectively; after 68 weeks, only 5% of the β-TCP remained (16). The degradation rate of TCP was higher than the new bone formation rate, which is likely to affect the repair of bone defects.

Growth factors are also important in bone tissue engineering and play critical roles in the regulation of osteogenesis, induction of osteogenic differentiation, cell proliferation, collagen synthesis and vascularization. PRP contains a variety of growth factors including platelet-derived growth factor (PDGF), transforming growth factor-β (TGF-β), insulin-like growth factor (IGF), vascular endothelial growth factor (VEGF) and epidermal growth factor (EGF) (17–19). As early as 1995, Slater *et al* revealed that PRP was able to promote the proliferation of osteoblasts *in vitro* (20). PRP may also promote BMSC differentiation and induction. It has been demonstrated that the differentiation capability of human BMSCs reduces as the cell passage number increases; however, the application of PRP substantially enhances the proliferation activity (21–23).

In the present study, PRP was successfully isolated from venous blood taken from the hind limb of a beagle dog using a two-step centrifugation procedure. The platelet concentration was ~8.6 times greater than that of whole blood. Bovine thrombin was added to the strengthened β-TCP/PRP gel composite in order to activate platelets to release growth factors. The gel composite extended the functional duration of the growth factors. Furthermore, the adhesion rates of the BMSCs were >90 and >95% at 12 and 24 h following implantation, respectively, although there was no significant difference compared with the control group. The MTT assay revealed that the toxic grade of the composite was zero based on the criteria used in the current study. The SEM micrographs demonstrated that the BMSCs adhered to the surface of the scaffold, proliferated and secreted ECM. Cellular proliferation and differentiation increased when an osteogenesis-directed differentiation inducer was added to the culture medium. The cell proliferation and secretion of ECM was extremely strong to the extent that the scaffold was almost completely covered and a three-dimensional structure was formed where the cells had gradually grown into the scaffold. However, the majority of the BMSCs were located on the surface of the scaffold, which may be associated with the preparation of the composite, cell concentration or the induction medium. Further study is required to gain further insight into the mechanism. The *in vivo* degradation behavior of the composite was not investigated in the current study and further study with an animal model is required to clarify the issue.

In the present study, a new composite combining strengthened β-tricalcium phosphate with PRP was successfully prepared with good biocompatibility and mechanical properties. The new composite may potentially be used as a scaffold to treat bone defects with bone tissue engineering technologies.

## Figures and Tables

**Table I:** Optical density (OD) and relative growth rate (RGR) values of bone marrow stromal cells in the control group and on the strengthened β-TCP/PRP composite.

	OD		
		
Day	Control group	Test group	RGR
2	0.172	0.173	1.007
4	0.242	0.257	1.064
8	0.951	0.968	1.017

TCP, tricalcium phosphate; PRP, platelet-rich plasma.
